# A Fuzzy-Based Model to Predict the Spatio-Temporal Performance of the *Dolichogenidea gelechiidivoris* Natural Enemy against *Tuta absoluta* under Climate Change

**DOI:** 10.3390/biology11091280

**Published:** 2022-08-28

**Authors:** Komi Mensah Agboka, Henri E. Z. Tonnang, Elfatih M. Abdel-Rahman, John Odindi, Onisimo Mutanga, Samira A. Mohamed

**Affiliations:** 1 International Centre of Insect Physiology and Ecology (ICIPE), Nairobi P.O. Box 30772-00100, Kenya; 2 School of Agricultural, Earth, and Environmental Sciences, University of KwaZulu-Natal, Pietermaritzburg 3209, South Africa

**Keywords:** fuzzy sets, crop pests, biological control, parasitoid, tomato farms

## Abstract

**Simple Summary:**

*Tuta absoluta* is an invasive pest threatening the productivity of the tomato crop. *Dolichogenidea gelechiidivoris* was imported and released as a natural enemy of *Tuta absoluta*. Mapping the efficacy of *Dolichogenidea gelechiidivoris* can improve its use as a control agent against *Tuta absoluta*. The *Dolichogenidea gelechiidivoris* efficacy map provides a tool for its targeted deployment as a *Tuta absoluta* natural enemy.

**Abstract:**

The South American tomato pinworm, *Tuta absoluta*, causes up to 100% tomato crop losses. As *Tuta absoluta* is non-native to African agroecologies and lacks efficient resident natural enemies, the microgastrine koinobiont solitary oligophagous larval endoparasitoid, *Dolichogenidea gelechiidivoris* (Marsh) (Syn.: Apanteles gelechiidivoris Marsh) (Hymenoptera: Braconidae) was released for classical biological control. This study elucidates the current and future spatio-temporal performance of *D. gelechiidivoris* against *T*. *absoluta* in tomato cropping systems using a fuzzy logic modelling approach. Specifically, the study considers the presence of the host and the host crop, as well as the parasitoid reproductive capacity, as key variables. Results show that the fuzzy algorithm predicted the performance of the parasitoid (in terms of net reproductive rate (R_0_)), with a low root mean square error (RMSE) value (<0.90) and a considerably high R^2^ coefficient (=0.98), accurately predicting the parasitoid performance over time and space. Under the current climatic scenario, the parasitoid is predicted to perform well in all regions throughout the year, except for the coastal region. Under the future climatic scenario, the performance of the parasitoid is projected to improve in all regions throughout the year. Overall, the model sheds light on the varying performance of the parasitoid across different regions of Kenya, and in different seasons, under both current and future climatic scenarios.

## 1. Introduction

In the last two decades, Africa has become the hotspot for many crop pest invasions. These include the *Bactrocera dorsalis* (Hendel) (Diptera: Tephritidae) in 2003 [1], *Tuta absoluta* (Meyrick) (Lepidoptera: Gelechiidae) in 2007 [2], *Spodoptera frugiperda* (J.E. Smith) (Lepidoptera: Noctuidae) in 2016 [3], and *Drosophila suzukii* Matsumura (Diptera: Drosophilidae) in 2019 [4]. The invasion by these devastating pests and the damage to their respective host plants have far-reaching economic and ecological implications. For example, following the detection and spread of the South American tomato pinworm, *Tuta absoluta*, up to 100% crop losses have been reported [5,6]. In addition to yield and monetary losses, environmental damage arises due to the heavy use of insecticides as a mitigation measure.

Significant crop loss caused by *T. absoluta* can be attributed to several factors that include the pest’s high dispersal capacity [7], wide thermal tolerance [8], high reproduction rate [8], lack of efficient co-evolved natural enemy to suppress its exploding population [9], and ability to adapt to the new ecosystems [10,11]. Due to enormous yield losses attributable to *T*. *absoluta* and its fast spread, farmers have generally adopted synthetic chemical insecticides as a primary mitigation measure [12]. However, this approach has diverse negative impacts on human, animal, and environmental health [13,14,15]. Furthermore, the approach is often ineffective in the management of the pest due to resistance to several classes of insecticides [15,16] and the cryptic nature of most of its developmental stages [17,18,19]. Therefore, an integrated management strategy in harmony with the environment is necessary for the sustainable management of *T. absoluta*. Being non-native to African agroecologies and lacking efficient resident natural enemies, classical biological control presents an ideal option for the suppression of the pest.

The International Centre of Insect Physiology and Ecology (ICIPE) in Nairobi, Kenya, jointly with the International Potato Center (CIP) in Lima, Peru, introduced the microgastrine koinobiont solitary oligophagous larval endoparasitoid, *Dolichogenidea gelechiidivoris* (Marsh) (Syn.: Apanteles gelechiidivoris Marsh) (Hymenoptera: Braconidae). The parasitoid was first imported from Peru to Kenya in 2017 for testing under quarantine conditions and final field release for classical biological control [9], based on its efficiency against *T. absoluta* in its aboriginal home range. For example, in Peru and Colombia, parasitism rates of about 57% [20] and 77% [21], respectively, have been reported.

Following its introduction in Kenya, and based on its outstanding performance against the African population of the target pest [9], the parasitoid was first released in Kirinyaga County, Kenya, in October 2020, as the first attempt for classical control of *T. absoluta* since its transatlantic invasion in 2006. However, its successful establishment and subsequent impact on the pest is determined by, among other things, environmental variables, particularly temperature [22] and the availability of the host insect [23,24,25] and host plants [26].

Using temperature and other climatic variables, several models have been proposed to project the habitat suitability for the establishment of natural enemies in Africa [8]. Indeed, these models have generated an important body of knowledge with regard to the potential parasitoid establishment. However, few of these models have investigated the natural enemy’s temperature-dependent performance at scale and linked it to the presence of the host and the host crop under present and future climatic conditions. Furthermore, the limitation of such models includes the uncertainties surrounding the complexity of the mechanism underpinning the establishment of parasitoids in different agroecologies, hence the need for more flexible and robust modelling approaches.

The use of the fuzzy set theory holds great potential in dealing with such uncertainties. Unlike other Artificial Intelligence approaches that include machine learning algorithms (maximum entropy and the genetic algorithm for ruleset prediction (GARP)), fuzzy sets allow for a representation of the inherent uncertainty in the data, as well as a linguistic description of the pattern under study, allowing for more accurate conclusions [27,28]. Furthermore, fuzzy sets are flexible and can be combined with other domain knowledge such as a geographic information system (GIS). Such combinations have been successfully used to provide insight into biological control agents’ mechanisms in managing insect pests at scale. For example, Bone et al. [29] used a combination of these approaches to identify areas vulnerable to the mountain pine beetle, *Dendroctonus ponderosae* Hopkins. Furthermore, Garcia et al. [30] augmented the work of Bone et al. [29] by developing an index to identify the most optimal areas to release *Tamarixia radiata* for the biological control of *Diaphorina citri.* This study exploits a fuzzy logic algorithm to determine the current and future spatio-temporal performance of *D. gelechiidivoris* against *T*. *absoluta* in tomato cropping systems, with a focus on the presence of the host and the host crop and the parasitoid reproductive capacity as key variables.

## 2. Materials and Methods

### 2.1. Study Area

**Figure 1:**
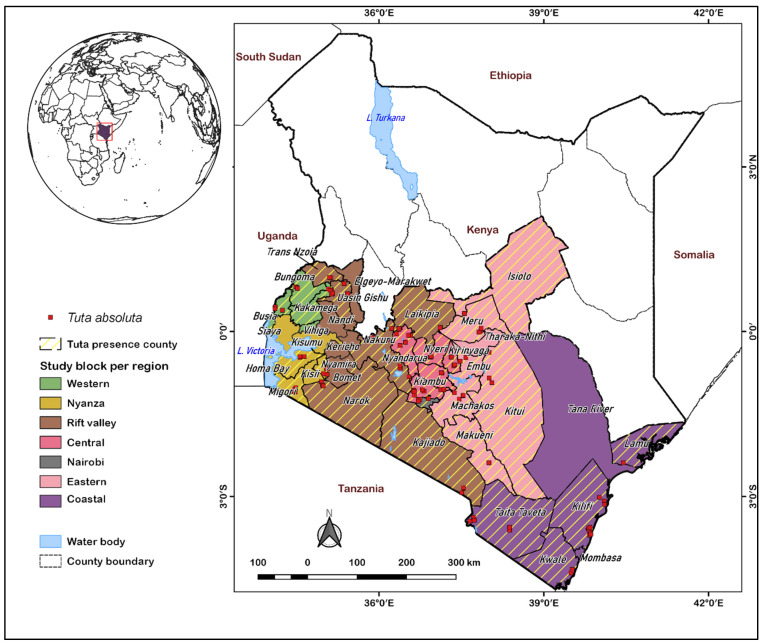
The study site showing the occurrence of the parasitoid host, *T. absoluta*.

The study was conducted in Kenya’s major tomato growing regions, i.e., 38 counties stretching through the Western, Nyanza, Eastern, Coast, Rift valley, and Central regions (Figure 1). The study sites represent all the agroecological zones, i.e., the humid, sub-humid, semi-humid, arid, and semi-arid zones. The regions exhibit two tomato cropping seasons dependent on rainfall patterns, with the long rainy season from March to July and the short rainy season from October to December. The temperature varies across the different tomato-growing regions (Table 1). Generally, most regions support various cropping systems, such as mono-cropping, mixed farming, and rotational farming systems, throughout the cropping calendar [31].

### 2.2. Assumption and Modelling Procedure

The fuzzy logic modelling approach used in this study integrates predefined rules and assumptions derived from the bioecology of the parasitoid and its host. The implementation and computation of the multiscale modelling approach were carried out in R statistical software [32]. The primary assumptions were (1) the parasitoid, *D. gelechiidivoris,* would survive within the same habitats where *T. absoluta* can survive; (2) the cropland contained *T. absoluta*’s primary (i.e., tomato) and secondary (other solanaceous) crops; (3) areas with a probability of greater than 0.2 were assumed to be suitable for *T. absoluta,* as the pest has been reported to be very resilient to a wide range of temperatures [9]; and (4) the parasitoid efficacy primarily depended on temperature; therefore, other climatic variables were set at optimal levels [33,34].

### 2.3. Data

The data used in modelling were related to the presence of the parasitoid host crop (tomato), the presence of the parasitoid host, *T*. *absoluta*, and the performance indicator of the parasitoid, i.e., the net reproductive rate (R_0_). For the presence of tomato in the study sites, Landsat 8 images (2020) were downloaded from the USGS portal https://earthexplorer.usgs.gov (accessed on 20 January 2022) and used to classify the study area’s land use/land covers (LULCs). The ground truth data for training and accuracy assessment were obtained in the Google Earth Engine (GEE) platform, while the presence of *T*. *absoluta* was confirmed using the MaxEnt ecological niche model. The georeferenced records used in the study were obtained from Kinyanjui et al. [35] and the 19 bioclimatic variables were sourced from the WorldClim platform www.worldclim.org (accessed on 20 January 2022) at an approximate 1 km^2^ spatial resolution [36,37]. The net reproductive rate (R_0_) was obtained from Aigbedion-Atalor et al. [38] and used as an indicator for parasitoid performance. In this regard, we defined the R_0_ as the average number of daughters that an adult female produces during her lifetime, which is one of the indicators of parasitoid performance on its host. For extrapolation over the study area under future scenarios, the Model Intercomparison Project of the Max Planck Institute Earth System Model Lower-Resolved version (MPI-ESM1.2-LR) projection was used due to its popularity [39]. The future scenario of the shared socioeconomic pathways version (SSP2-4.5) was hypothesized as the more realistic case scenario under the Coupled Model Intercomparison Project Phase 6 (CMIP6).

### 2.4. Land Use/Land Cover Characterization

Satellite image pre-processing, classification and accuracy assessment, and post-classification processing were used to determine the study area’s LULC. Firstly, cloud-free Landsat imagery was pre-processed to correct for atmospheric anomalies [40,41]. The images were then projected to Google Mercator (EPSG: 3857) and clipped according to the study area boundary. The individual bands were stacked and displayed as standard false colors (4-3-2 for TM and for 5-4-3 for OLI) for vegetation analysis.

Secondly, training samples were automatically selected in the GEE platform for classification and accuracy assessment. The selected samples were then used to extract Landsat 8 spectral and temporal features to train the random forest classifier. Details of the nine delineated LULC classes are given in Table S1 (supplementary). Furthermore, image validation was carried out using high resolution Google Earth images. The digitized polygons were then converted to raster format using the rasterize tool in QGIS, and the r.kappa tool in QGIS was used to compare classified outputs and rasterized referenced data. Finally, the classified outputs were filtered using a majority filter to remove noise and enhance cartographic appearance for post-classification processing. The LULC statistics were obtained for each class value using the raster layer unique value statistics tool in QGIS. The result was then imported into Excel and converted to square kilometres (km^2^) (Table S2 in supplementary). The error matrix, accompanied by statistical measures such as overall accuracy (OA) and kappa (K) coefficient (Table S3 in supplementary), was generated and assessed.

### 2.5. Presence of the Parasitoid Host, T. absoluta

MaxEnt software version 3.4.1 [42] was used to predict the habitat suitability of the parasitoid host, *T. absoluta,* for the classified LULCs in the study area. The georeferenced records of *T. absoluta* were obtained from Kinyanjui et al. [35] and the 19 bioclimatic variables were sourced from the WorldClim platform www.worldclim.org (accessed on 20 January 2022) [36,37], in addition to the classified LULCs used as variables. Prior to running MaxEnt, the variables were clipped to Kenya country boundaries and converted to American Standard Code for Information Interchange (ASCII) format using the ‘raster’ package [43] in R statistical software [32]. Then, the ‘virtual species’ package [44] in R statistical software [32] was used to explore the spatial correlation of the 19 bioclimatic variables using the Pearson’s correlation coefficient and the cluster tree. A 0.7 threshold cutoff of the Pearson’s correlation coefficient was used to select the variables (Figure S1 in supplementary). The least-correlated bioclimatic variables were then consolidated with other predictor variables in the MaxEnt model (Table S4 in supplementary). The predictor variables had different spatial resolutions, and hence, they were resampled to the highest resolution in the dataset (30 m pixel size). The MaxEnt model was run in 5000 iterations and five replicates for better accuracy, and the performance of the MaxEnt model was assessed using the area under the curve (AUC).

### 2.6. Predicting the Spatio-Temporal Performance of D. gelechiidivoris

The spatio-temporal performance of *D. gelechiidivoris* at scale was predicted for each month of the year to include pre- and post-tomato cropping for current and future projection. To predict the spatio-temporal, temperature-based performance of *D. Gelechiidivoris* on its host, *T. absoluta*, we automated deduction through computation using the rules described in Table 2 as the main component. The Mamdani inference fuzzy algorithm theory was preferred due to its simplicity and popularity [45]. These rules were obtained by expert knowledge [46] using laboratory findings on the temperature-based performance of the parasitoid, i.e., the parasitoid net reproductive rate (R_0_) (Table S5 in supplementary). The algorithm entails the following fundamental rules: IF x is *M* THEN y is *N* [45]. For the semantical interpretation extended to the set [0,1] of truth values [47] of IF x is *M* THEN *y* is *N* in the Generalized Modus Ponens (GMP), we chose a fuzzy relation (M → N)(x, y)=min(M(x),N(y)) and we computed $N^{\prime }$ in the GMP as follows [48]:

(1) $$N^{\prime }\left(y\right)=\left(M^{\prime }\circ \left(M\to N\right)\right)\left(y\right)=\underset{x\in X}{\vee }\left(M^{\prime }\left(x\right)\wedge N\left(\;y\right)\right),\;y\in Y.\;$$

where $M^{\prime }$ and $N^{\prime }$ denote restrictions related to M and N.

Overall, we considered and extended the conditional premise of the Mandani GMP to be valid for each *i* = 1,…,n [48] rule defined for the parasitoid performance, as follows:

(2) $$rules_{i}=IF\;x\;is\;M_{i}\;THEN\;y\;is\;N_{i},\;i=1,\;\ldots n$$

where $M_{i}$ represents fuzzy sets (the parasitoid temperature-based performance), x is the premise variable (temperature in °C), and y is the consequence variable, i.e., the performance of the parasitoid (R_0_). To assess the performance of the Fuzzy logic algorithm, the root mean square error (RMSE) and the coefficient of determination (R^2^) were used as the accuracy metrics.

## 3. Results

The error matrix showed very high classification accuracy (i.e., overall accuracy of 0.9, with a kappa coefficient (K) of 0.92), suggesting a reliable LULC classification. The classification output revealed that a significant (50.28%) proportion of the study area is dominated by grass cover, followed by cropland (37.61%), and barren class (0.05%) (Table S2 in supplementary). Furthermore, the mapping of the LULC revealed that croplands are primarily located in the key tomato production counties (Figure S2 in supplementary).

The MaxEnt modelling algorithm showed an average AUC of greater than 0.85, indicating that the model accurately predicted the distribution of *T. absoluta*. The results confirmed a high risk of infestation of *T. absoluta* in the selected study sites (Figure S3 in supplementary).

**Figure 2:**
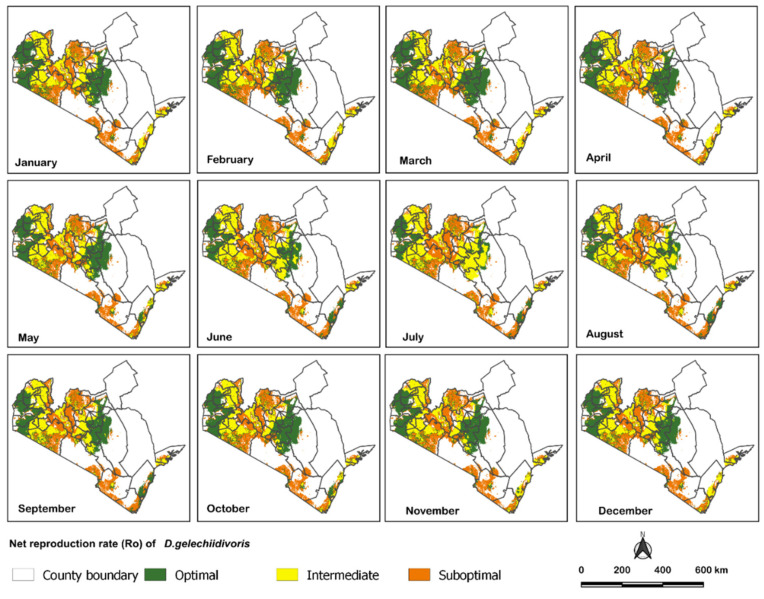
Spatio-temporal performance of *D. gelechiidivoris* in the strategic tomato production areas under the current climate conditions.

**Figure 3:**
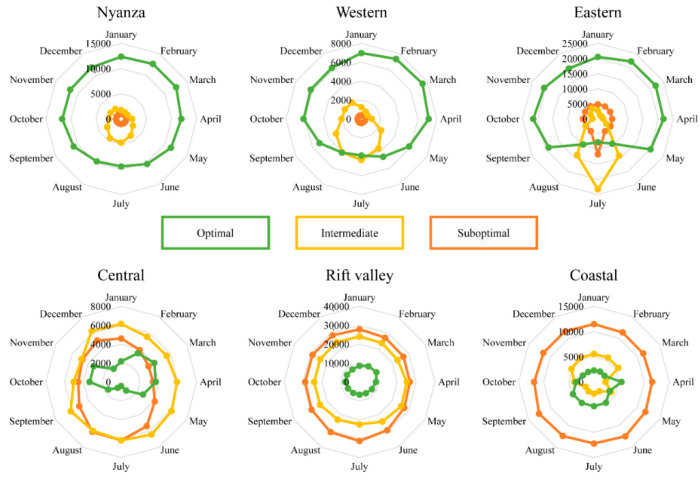
Current projection of the performance of *D. gelechiidivoris* in selected regions in Kenya using pixels counts (the number of pixels in each class per month).

The fuzzy algorithm predicted the performance of the parasitoid (R_0_) with a low RMSE value (<0.90) and a considerably high R^2^ (=0.98) coefficient, accurately predicting the parasitoid’s spatio-temporal performance. Under the current climatic scenario, the parasitoid is predicted to perform fairly well in all regions throughout the year, except for the coastal region (Figure 2). The outcome of the fuzzy algorithms in predicting the parasitoid spatio-temporal distribution is further substantiated by the number of pixels of each class extracted using QGIS [49]. In two regions (i.e., Nyanza and Western), where the parasitoid was projected to perform optimally, the performance did not vary considerably across the seasons (Figure 3). On the other hand, the parasitoid performance was predicted to be worse in the Rift valley and very poor in the coastal regions. Interestingly, the parasitoid exhibited an erratic yet fairly good performance in the Central region across the seasons, with July and August being the least conducive for a parasitoid (Figure 3).

**Figure 4:**
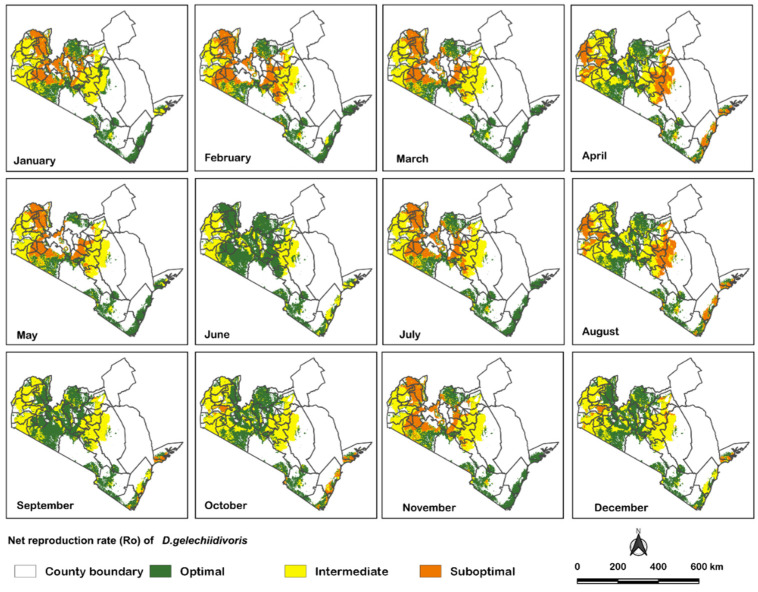
Spatio-temporal performance of *D. gelechiidivoris* in the strategic tomato production areas under the future climate projection (2050).

**Figure 5:**
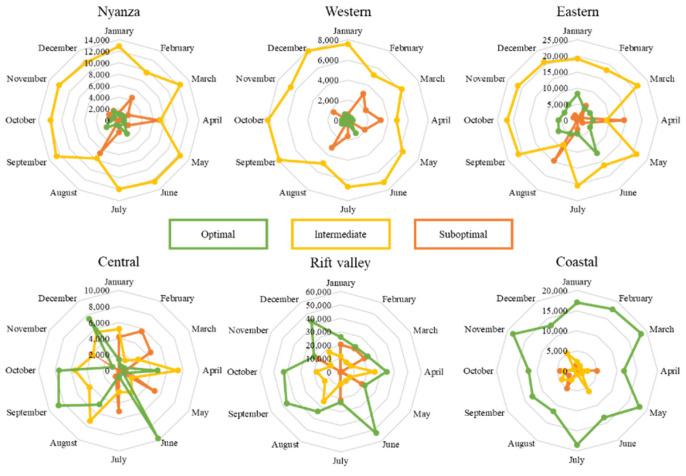
Future (2050) projection of the performance of *D. gelechiidivoris* in selected regions in Kenya using pixels counts (the number of pixels in each class per month).

Under the future climatic scenario, overall, the performance of *D. gelechiidivoris* was projected to improve (Figure 4). Nevertheless, the parasitoid performance in the Nyanza and Western regions was predicted to be good, rather than optimal, across the year. Conversely, in the Rift valley and coastal regions, the parasitoid performance was predicted to improve considerably as compared to the current scenario, being optimal for the entire year. Notably, the parasitoid performance remained erratic in the Central region, but with an overall noticeable enhancement. In particular, the parasitoid performance was projected to be enhanced considerably in June and December. On the other hand, the parasitoid performance was projected to slightly decline in February and March compared to the current scenario (Figure 5).

## 4. Discussion

Alien invasive insect pests commonly arrive at their new invasive range without their efficient, co-evolved natural enemies; hence, they tend to multiply exponentially, with far-reaching socioeconomical and ecological consequences. This is similar to the South American tomato leafminer, *T*. *absoluta,* in its Afro-Eurasian invasion. In Kenya, although several indigenous parasitoid species have been reported to be associated with the pest, their performance in terms of percent parasitism is quite low [35]. On the other hand, the laboratory performance of the introduced *D. gelechiidivoris’s* percent parasitism [9] and density-dependent response [50] makes it a promising biocontrol approach for *T*. *absoluta*.

Although the laboratory experiments provide vital information on parasitoid performance, the findings may not necessarily mirror its performance under natural field conditions. Indeed, in nature, the parasitoid performance against its target host is governed by an array of complex and intertwining biotic and abiotic factors, which can impact negatively or positively on the outcome of biological control (e.g., [51,52,53]). These factors include parasitoid reproductive rate and dispersal ability, inter- and intra-specific competition, predation and availability of host insect, and the associated host plants [33,34]. Other factors include parasitoid thermal tolerance [33,34] and farmers’ management practices such as the use of pesticides and soil health management within the landscape matrix [54].

Various ecological and phenological models have recently been used to predict the establishment of different parasitoid species under current and/or future scenarios in Africa [8]. Using Insect Life Cycle Modelling (ILCYM) software, the thermal thresholds and demographics parameters of *D. gelechidivoris* have been investigated based on the thermal response curves [38]. Indeed, these models have generated substantial knowledge regarding the potential parasitoid establishment. Nevertheless, none of these models has addressed the aspects related to parasitoid spatio-temporal distribution. Additionally, none of these models has considered parameters such as LULC and the host insect availability. Furthermore, these models have some limitations which include the uncertainties surrounding the complexity of the mechanism underpinning the establishment of parasitoids in different agroecologies; hence, there is a necessity for more flexible modelling approaches.

In the current study, using a more robust yet flexible modelling approach that combined the fuzzy logic model and the species distribution model, we incorporated three key variables, namely, the availability of the host insect and its associated host plants as mapped by LULC and the temperature-based performance of the parasitoid in terms of the parasitoid net reproductive rate (R_0_), as reported by Aigbedion-Atalor et al. [38]. The approach was used to model the spatio-temporal performance of the *T. absoluta* parasitoid, *D. gelechiidivoris,* in vulnerable regions of Kenya. The outcome of the fuzzy logic model predicted the performance of *D. gelechiidivoris* with very high accuracy (R^2^ = 0.98). The spatio-temporal differential performance of the parasitoid across the various regions could be explained by the different climatic conditions that characterize these regions, with the Rift valley and coastal regions being too cold and too hot, respectively, for *D. gelechiidivoris* (Table 1). Similar findings for varying performance with season across the different regions in Kenya were reported for other parasitoid species. For example, *Diglyphus isaea* (Walker) (Hymenoptera: Eulophidae) was reported to be more abundant at the high and middle elevations throughout the year, while *Phaedrotoma scabriventris* (Nixon) (Hymenoptera: Braconidae) and *Opius dissitus* Muesebeck (both (Hymenoptera: Braconidae) had better performances at low elevations during the long rainy season [55].

With the expected temperature increase of 2 °C by the year 2050, the parasitoid performance against the target pest, *T. absoluta*, is generally predicted to improve, notably in the Rift valley and coastal regions. In the Rift valley region, this enhancement in performance could be attributed to an increase in temperature to an optimal range of 20–25 °C. However, in the coastal region, the reason for enhanced performance is unclear. We speculate that the reason for enhanced performance could be attributed to the fact that the target pest population may be reduced considerably due to potential stressors.

Nevertheless, we recognize the importance of incorporating additional information pertaining to farmers’ practices. For instance, a model incorporating the frequency of pesticide usage and its effect on parasitoid performance should lead to a more refined prediction. It was reported that farmers in Kirinyaga county are spraying up to 16 times per tomato growing season, which reduces the abundance of natural enemies [56]. In addition, a layer’s resolution impacts the output accuracy when using a geospatial algorithm [57]; therefore, since the study targeted small-scale farmers, using higher-resolution bioclimatic data, the same as the classified LULC (i.e., a 30 m resolution), will increase the accuracy of the results. Nevertheless, the outcome of our model provides a solid ground and essential information to guide the parasitoid release and augmentation at both temporal and spatial scales within *T*. *absoluta’s* framework of the Integrated Pest Management (IPM). A similar conclusion was drawn by Bone et al. [29], who confirmed that the fuzzy sets theory could satisfactorily be used as a tool for decision support regarding insect pest management. Furthermore, Garcia et al. [30] acknowledge the use of fuzzy sets to develop realistic models regarding biological systems at a landscape level.

Despite the fuzzy algorithm’s high accuracy in the present study, it is important to highlight that this algorithm suffers from a few limitations and drawbacks, as does any other Artificial Intelligence algorithm. For instance, fuzzy logic is a bit generic, and its inputs are somewhat not precisely defined in most study cases [46]. In other words, the fuzzy logic inputs are largely defined by expert knowledge. Hence, these inputs might not be exactly the same (i.e., they could vary considerably according to the expert understanding of the system). For example, in this study experiment, we defined the presence of the parasitoid at a 0.2 level of probability. However, for another case study, such a parasitoid presence could be defined at a 0.4 probability score. Therefore, special care should be taken before extrapolating the model developed in this study to other points in space and time, as the choice of a model usually depends on the study’s purpose and available information [58,59]. However, there are advantages that fuzzy logic offers in terms of flexibility and simplicity, which reduce the model’s vulnerability to uncertainty as no exact information on the system is needed [46]. Given these advantages, the fuzzy logic model is one of the most useful Artificial Intelligence algorithms in many agricultural applications, and its importance is growing considerably in ecology with successful applications in insect pest management (e.g., [29,60]) and biological control [30]. For example, Center and Verma [61] described the applications of fuzzy logic in biological and agricultural systems. They concluded that fuzzy logic provides a methodology for describing complex systems and performs better than the conventional (parametric) methods for time-varying, nonlinear, adaptive systems, such as those found in biological and agricultural processes. Such advantages make it suitable for this study, as demonstrated by the high accuracy obtained.

## 5. Conclusions

This study predicted the spatio-temporal performance of *D. gelechiidivoris* as a biocontrol agent for *T. absoluta* using fuzzy sets through a multilevel modelling approach, with high accuracy. The fuzzy logic modelling approach used in this study integrates predefined rules and assumptions derived from the bioecology of the parasitoid and its host. The data used in modelling were related to the presence of the parasitoid host crop (tomato), the parasitoid host, *T*. *absoluta*, and the performance indicator of the parasitoid. The outcome of the fuzzy logic model predicted the performance of *D. gelechiidivoris* with very high accuracy (R^2^ = 0.98). Under the current climatic scenario, the parasitoid is predicted to perform fairly well in all regions throughout the year, except for the coastal region. Under the future climatic scenario, the performance of *D. gelechiidivoris* is projected to improve. Overall, the model sheds light on the varying performance of the parasitoid across different regions of Kenya and in different seasons under both current and future climatic scenarios. The outcome of our model provides solid ground and the essential information to guide the parasitoid release and augmentation at both temporal and spatial scales within *T*. *absoluta’s* IPM framework.

## Figures and Tables

**Table 1:** Monthly long-term average temperatures (1991–2021) per tomato cropping region.

Months ^1^	Western	Nyanza	Rift Valley	Central	Eastern	Coast
January	21.0	23.6	18.9	19.3	19.6	27.9
February	21.9	24.6	19.5	20.0	20.5	28.1
March	21.6	23.9	19.3	19.8	20.4	28.7
April	20.5	22.8	18.1	18.6	19.5	28.1
May	19.5	22.5	17.2	17.7	18.7	26.6
June	18.6	22.4	16.4	16.8	17.8	25.9
July	18.2	22.5	16.1	16.3	17.2	25.4
August	18.3	22.7	16.5	16.7	17.6	25.5
September	19.0	23.2	17.5	17.9	18.8	26.2
October	19.7	23.2	18.3	18.6	19.6	26.8
November	19.8	22.7	17.7	17.8	18.7	27.2
December	20.2	22.9	18.1	18.2	18.7	27.7

^1^ Source: Climate-data.org https://en.climate-data.org/ (accessed on 20 February 2022).

**Table 2:** Net reproduction rate (R_0_) of *D. gelechiidivoris* based on temperature variability.

Temperature Threshold	Net Reproduction Rate (R_0_)	Fuzzy Partition Variable Names
10–15 °C	0.13–1.55	Suboptimal lower temperature threshold
20 and 25 °C	15–14	Optimal
30–35 °C	2.18–0.06	Suboptimal higher temperature threshold

## Data Availability

All data generated or analyzed during this study are included in this published article (and its supplementary information files). The codes that support the findings of this study are available permanently and freely online at the International Centre of Insect Physiology and Ecology (ICIPE) data warehouse through the following link: https://dmmg.icipe.org/dataportal/dataset/a-fuzzy-based-modelling-approach-to-predict-the-spatio-temporal-performance (accessed on 10 August 2022).

## Supplementary Materials

The following supporting information can be downloaded at: https://www.mdpi.com/article/10.3390/biology11091280/s1, Figure S1: The least-correlated bioclimatic variables’ spatial correlation with the selected variables highlighted with red; Figure S2: Classified LULC map for different years based on the nine classes extracted from the study area with a proportionate coverage area; Figure S3: Map of suitability areas of *T. absoluta* for the classified LULC in the strategic tomato production areas; Table S1: Description of the LULC classes of the study; Table S2: Landcover statistics; Table S3: Accuracy assessment; Table S4: Selected non-colinear variables used to predict the habitat suitability for *Tuta absoluta* using the MaxEnt model; Table S5: Temperature-based performance of the parasitoid *Dolichogenidea gelechiidivoris*.
