# Supplementary material for: A Fuzzy-Based Model to Predict the Spatio-Temporal Performance of the Dolichogenidea gelechiidivoris Natural Enemy against Tuta absoluta under Climate Change
Source: Biology (Basel). 2022 Aug 28;11(9):1280. doi: 10.3390/biology11091280 (PMC9495800; doi:10.3390/biology11091280)
Supplement: Supplementary file 1 [file biology-11-01280-s001.zip › biology-1842183-SI.pdf]

biology
MDPI

Supplementary

Table S1. Description of LULC classes of the study

Land Cover	Description
**Barren**	Lands with exposed soil, sand, or rocks and never has more than 10% vegetated cover during any time of the year.
**Built up**	Land covered by buildings and other manmade structures.
**Cropland**	Lands covered with temporary crops followed by harvest and a bare soil period (e.g., Single and multiple cropping systems).
**Forest**	Refer to land with a tree canopy cover of more than 10 percent.
**Grassland**	Dominated by herbaceous annuals (<2m).
**Savanna**	Tree cover 10-30% (canopy >2m).
**Shrubs**	Woody perennial plants with persistent and woody stems and without any defined main stem being less than 5 metre tall. The shrub foliage can be either evergreen or deciduous.
**Water**	At least 60% of area is covered by permanent water bodies.
**Wetlands**	Permanently inundated lands with 30-60% water cover and >10% vegetated cover.

Table S2. Landcover statistics

Classes	Area (km2)	Proportion %
**Forest**	5735.1	2.02
**Shrubs**	5523.46	1.94
**Savanna**	14387.02	5.06
**Grassland**	143013.82	50.28
**Wetlands**	973.18	0.34
**Cropland**	106978.82	37.61
**Built up**	404.5	0.14
**Barren**	144.47	0.05
**Water**	7300.7	2.57
**Total**	**284461.06**	**100**

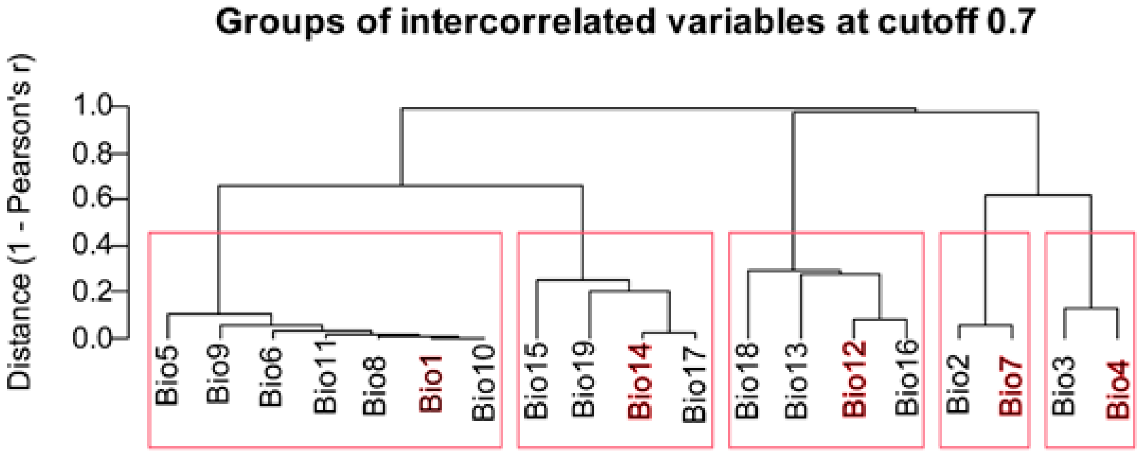

Figure S1. The least correlated bioclimatic variables spatial correlation with selected variables highlighted with red.

Table S3. Accuracy assessment

	Reference									
**Cat**	Forest	Shrubs	Savanna	Grassland	Wetlands	Cropland	Built up	Barren	Water	Row Sum
**Forest**	28958	0	509	0	0	51	0	0	0	29518
**Shrubs**	0	4940	0	8714	0	176	0	0	0	13830
**Savanna**	215	0	18764	456	0	133	0	0	0	19568
**Grassland**	0	0	2056	20033	0	34	0	0	0	22123
**Wetlands**	0	0	550	0	1130	42	0	0	0	1722
**Cropland**	0	0	0	0	0	81562	0	0	0	81562
**Built up**	0	0	0	0	0	85	2411	0	0	2496
**Barren**	0	0	0	524	227	0	0	885	0	1636
**Water**	0	0	0	0	277	0	0	0	71767	72044
**Col Sum**	29173	4940	21879	29727	1634	82083	2411	885	71767	244499
			Kappa	0.92						
			Overall Accuracy	94.25						

Table S4. Selected non-colinear variables used to predict the habitat suitability for *Tuta absoluta* using the MaxEnt model.

Selected variable	description	Unit
**Bio1**	Annual Mean Temperature	°C
**Bio4**	Temperature Seasonality (standard deviation ×100)	°C
**Bio7**	Temperature Annual Range (BIO5-BIO6)	°C
**Bio12**	Annual Precipitation	mm
**Bio14**	Precipitation of Driest Month	mm
**LULC**	Land use land cover	Categorical

Table S5. Temperature-based performance of the parasitoid *Dolichogenidea gelechiidivoris.*

Temperature (◦C)	Net reproductive rate (R_0)
**10**	0.13
**15**	1.55
**20**	14.99
**25**	14.25
**30**	2.18
**35**	0.06

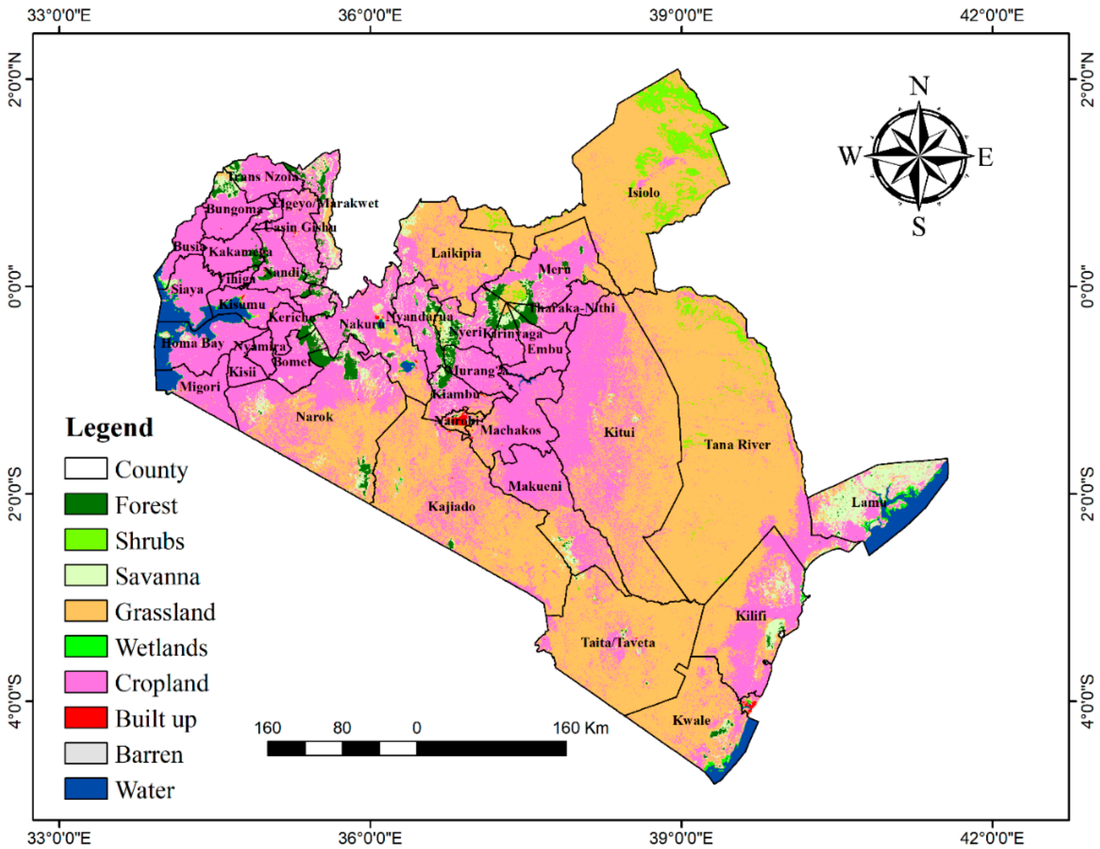

Figure S2. Classified LULC map for different years based on nine classes extracted from the study area with a proportionate coverage area.

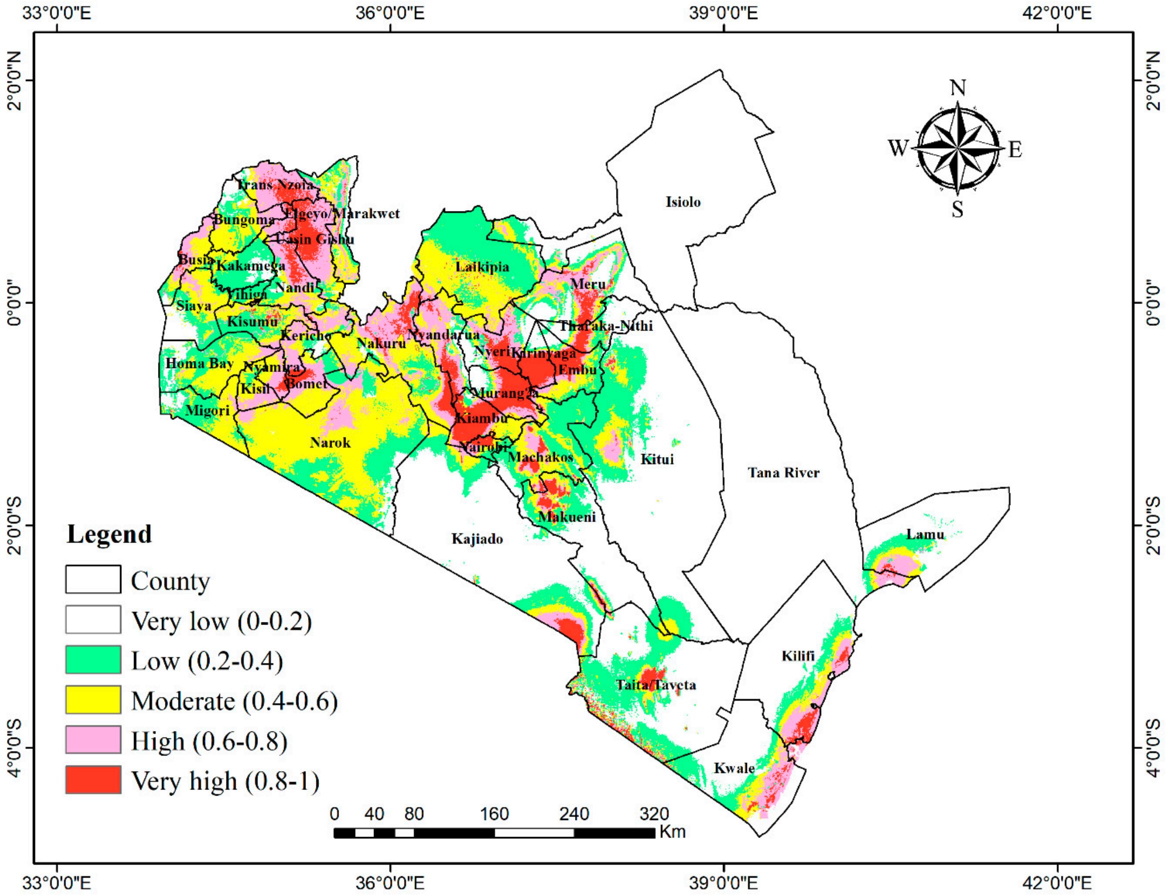

Figure S3. Map of suitability areas of *T. absoluta* for the classified LULC in the strategic tomato production areas.
